# Crack Protective Layered Architecture of Lead-Free Piezoelectric Energy Harvester in Bistable Configuration

**DOI:** 10.3390/s20205808

**Published:** 2020-10-14

**Authors:** Ondrej Rubes, Zdenek Machu, Oldrich Sevecek, Zdenek Hadas

**Affiliations:** Faculty of Mechanical Engineering, Brno University of Technology, Technicka 2896/2, 616 69 Brno, Czech Republic; Zdenek.Machu@vutbr.cz (Z.M.); sevecek@fme.vutbr.cz (O.S.); hadas@fme.vutbr.cz (Z.H.)

**Keywords:** energy harvesting, piezoelectrics, bimorph, lead free ceramic, bistable energy harvester, nonlinear resonators

## Abstract

Kinetic piezoelectric energy harvesters are used to power up ultra-low power devices without batteries as an alternative and eco-friendly source of energy. This paper deals with a novel design of a lead-free multilayer energy harvester based on BaTiO_3_ ceramics. This material is very brittle and might be cracked in small amplitudes of oscillations. However, the main aim of our development is the design of a crack protective layered architecture that protects an energy harvesting device in very high amplitudes of oscillations. This architecture is described and optimized for chosen geometry and the resulted one degree of freedom coupled electromechanical model is derived. This model could be used in bistable configuration and the model is extended about the nonlinear stiffness produced by auxiliary magnets. The complex bistable vibration energy harvester is simulated to predict operation in a wide range of frequency excitation. It should demonstrate typical operation of designed beam and a stress intensity factor was calculated for layers. The whole system, without presence of cracks, was simulated with an excitation acceleration of amplitude up to 1g. The maximal obtained power was around 2 mW at the frequency around 40 Hz with a maximal tip displacement 7.5 mm. The maximal operating amplitude of this novel design was calculated around 10 mm which is 10-times higher than without protective layers.

## 1. Introduction

Many energy harvesting devices have been developed in recent years in order to provide autonomous source of energy for autonomous IoT applications. A piezoelectric transducer is widely discussed for electro-mechanical conversion of common motion into the useful electricity [1]. A wide range of piezoelectric materials has been studied enormously for energy harvesting applications [2]. The most popular materials are lead zircon titanite PZT, BaTiO_3_, and like polyvinylidene fluoride (PVDF). The recent development is forced to focus on lead free ceramic materials and composites for energy harvesting devices [3]. Mainly, barium titanite ceramics are discussed in the case of lead-free applications [4]. Piezoelectric properties of the lead-free ceramic materials, e.g., BCZT ceramics [5], are sensitive to fabrication and processing methods. Alternatives to ceramic materials are provided by polymers like polyvinylidene fluoride (PVDF) [6].

Designs of kinetic energy harvesting devices for various applications were developed by many researchers [7]. Their desing depends on specific requirements and specific conditions where energy harvesting devices operate [8]. The piezoelectric energy harvesters were developed for many engineering applications ranging from biomedical devices [9] and wearable electronic devices [10] to mobile electronics and self-powered wireless network nodes, e.g., in aircraft applications [11].

A cantilever design of a piezoelectric energy harvesting system [12] in operational mode 31 is commonly used for kinetic energy harvesting devices. A model of piezoelectric cantilever structure can be used successfully for development of a specific design of energy harvesting system. The cantilever resonator commonly operates in a nonlinear manner but for a specific operation it can be linearised [13]. For a strong nonlinearity a model of Duffing energy harvester [14] could be used.

Non-linear vibration energy harvesting technologies with bistable operation were many times published and discussed. This well-known approach utilizes additional magnetoelastic forces to provide additional stiffness into the design structure of cantilever-based energy harvesters [15]. Several topologies of magnetic systems [16] can be used in the form of nonlinear stiffness force to design nonlinear vibration energy harvesting systems with one degree of freedom. Also, other concepts with two degrees of freedom design are feasible, e.g., the design published in [17]. However, a well-known concept used, consisting of two magnets, creates mainly wider bandwidth [18], lower operation frequency, and there is also the potential for chaotic operation in coloured noise excitation, e.g., [19,20].

**Figure 1:**
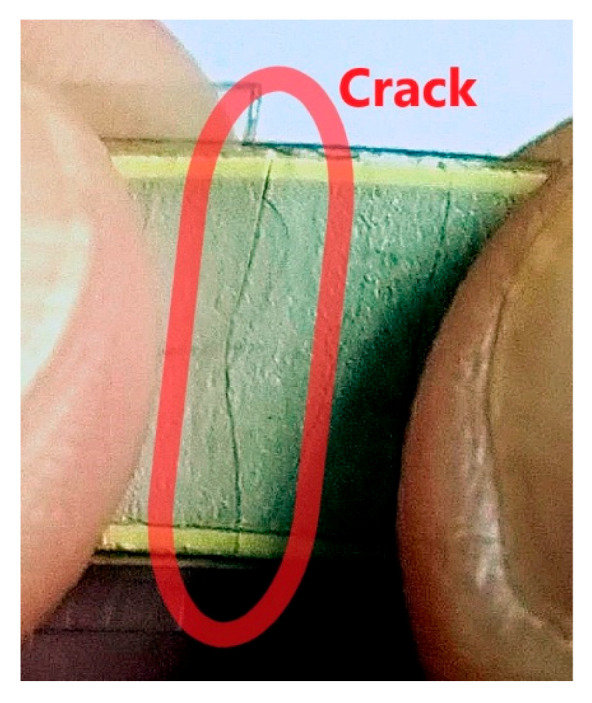
A crack in piezoceramic layer due to operation in high oscillation amplitude (@ 4 mm).

Mainly, bistable piezoelectric energy harvesters provide a strong nonlinearity in displacement in a range of few millimetres [21]. Nevertheless, piezoceramic energy harvesters also have certain limitations concerning short lifetime because since piezoelectric ceramics are brittle [22]. Cracking of a piezoceramic layer due to an operation in high oscillation displacement (@ 4 mm) which was tested for wearable energy harvesting [10] with highlighted crack is shown in Figure 1. In this case of very high displacement, PVDF materials are usually used [23] due to their flexibility. However, piezoceramic materials have a higher d_31_ coefficient which is a key for an efficient energy harvesting; in case of lead-free solution BaTiO_3_ this d_31_ coefficient is more than two times higher than the one of PVDF.

The main aim of this paper is to present a concept of a novel, layered architecture of the lead-free BaTiO_3_ piezoceramic beam with protective layers for bistable operation of piezoelectric energy harvesters, which provide a maximal peak to peak displacement around 20 mm without a brittle fracture. This large oscillation range of bistable energy harvester allows to generate a much higher output power.

## 2. Bimorph Cantilever Beam Design

**Figure 2:**
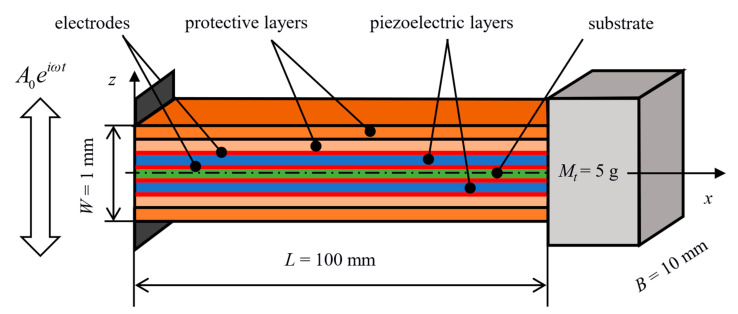
The considered multilayer piezoelectric harvester.

A bimorph configuration of a proposed piezoelectric energy harvester in the form of a geometrically symmetric multilayer ceramic structure is shown in Figure 2. This novel structure integrates protective ceramic layers on the outside of piezoelectric layers. The layers within the multilayer structure contain high thermal residual stresses which are induced upon manufacturing of the laminate due to a mismatch of thermal expansion coefficients of used materials. The basic idea of the multilayer design was firstly presented in the previous author’s work [24]. Unlike the traditional bimorph configuration (piezo–substrate–piezo) which is very prone to a brittle failure caused by unstable surface crack growth when excessively loaded, such a multilayer design with proper distribution of thermal residual stresses within the layers leads to a significant increase in the resistance of structure to surface crack propagation as presented in publications [25,26,27]. The residual stresses allow the multilayer harvester to withstand significantly higher mechanical loads (than traditional harvester designs) and simultaneously thus increase also the amount of generated electrical power.

As shown in Figure 2, the considered multilayer structure consists of seven layers: a substrate in the middle, a piezoelectric layer on each side of the substrate, followed by two protective layers. This number of layers was chosen to allow for a design of a relatively thin structure which is as compliant as possible. Dimensions of the structure were chosen to be *L* = 100 mm, *B* = 10 mm, and *W* = 1 mm. The free end also bears a tip mass *M_t_* = 5 g. The piezoelectric layers are considered to be made from BaTiO_3_ and are electrically connected in parallel. The substrate together with protective layers are considered to be made from ZrO_2_ and ATZ (Alumina Toughened Zirconia). These materials were chosen due to their relatively close values of thermal expansion coefficient *α* and, as a consequence, moderate levels of thermal residual stresses *σ_res_* are induced within the considered multilayer structure upon its fabrication. Material properties of the considered materials are listed in Table 1.

### 2.1. Optimization of the Layer Configuration within the Proposed Multilayer Structure

Individual layers of the multilayer structure need to have optimal thicknesses and materials without changing the total thickness of the structure in order to make the structure more resistant to a brittle failure via reasonably high levels of thermal residual stresses (induced upon the laminate fabrication). The aim of the optimization is to make the outer protective layers highly resistant to surface crack propagation to prevent potential surface cracks from expanding into the BaTiO_3_ layer, and thus causing a malfunction of the system. At the same time, BaTiO_3_ layers should be as thick as possible to minimise their capacitance so that the amount of generated electrical power is not suppressed.

Optimal thickness of each layer and sequence of used materials was sought similarly as in [24] to achieve both a reasonably low level of residual stresses *σ_res_* within the layers and at the same time to obtain the highest possible resistance to surface crack propagation quantified with the so-called apparent fracture toughness [30]. The apparent fracture can be effectively calculated by employing the weight function method described in [31], evaluating the apparent fracture toughness using the distribution of thermal residual stresses within the layers and a so-called weight function. Upon calculation, the crack path is assumed to not be affected by thermal residual stresses (i.e., the crack is assumed to grow perpendicular to the structure’s surface). Thermal residual stresses *σ_res_*, which are required for determining the apparent fracture toughness, can be quantified within *i*-th layer using relations from classical laminate theory [32] as:

(1) $$\sigma_{res,i}=\frac{E_{i}}{1-\nu_{i}}\left(\bar{\alpha }-\alpha_{i}\right)\Delta T,\;\mathrm{where}\;\bar{\alpha }=\sum_{i=1}^{N}\frac{E_{i}\alpha_{i}h_{i}}{1-\nu_{i}}/\sum_{i=1}^{N}\frac{E_{i}h_{i}}{1-\nu_{i}}$$

Here, *E_i_*, *ν_i_*, and *α_i_* is the elastic modulus, Poisson’s ratio and thermal expansion coefficient of the *i*-th layer respectively and Δ*T* is the temperature difference between the room and the zero-strain (reference) temperature. The term $\bar{\alpha }$ represents the apparent thermal expansion coefficient of the whole laminate and *h_i_* is the thickness of the *i*-th layer. The distribution of *σ_res_* is assumed to not be affected by piezoelectric properties of BaTiO_3_ layers. The apparent fracture toughness *K_R,eff_* can then be calculated using the weight function approach [31] as:

(2) $$K_{R,eff}\left(a\right)=K_{c,0}-\int_{0}^{a}h\left(z,a\right)\sigma_{res}\left(z\right)dz$$

where *K_c,_*_0_ is the intrinsic fracture toughness of current layer, *a* is the crack length, and *h*(*z*, *a*) is a weight function defined, e.g., in [33].

**Figure 3:**
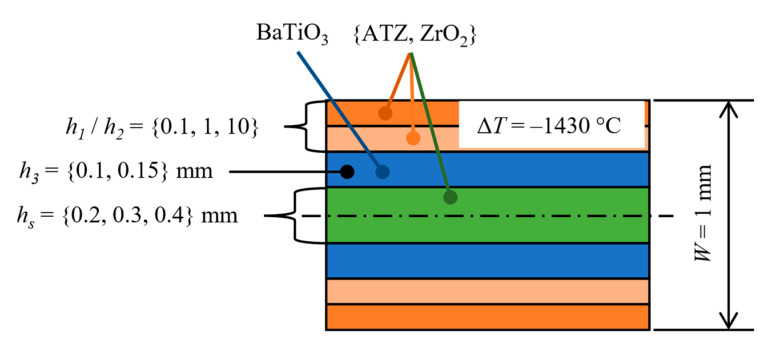
Scheme of the first optimization phase.

**Figure 4:**
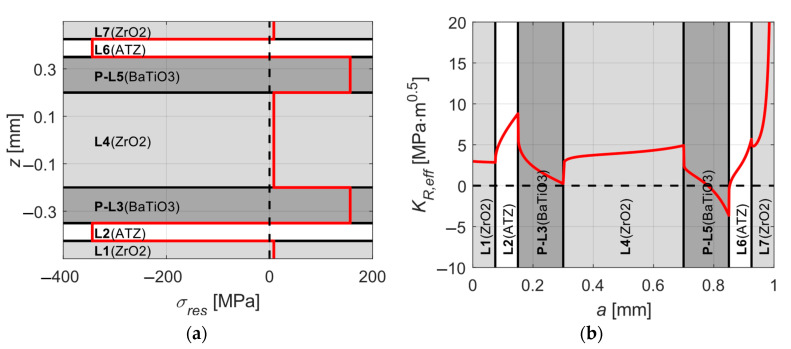
A ZrO_2_–ATZ (Alumina Toughened Zirconia)–BaTiO_3_ composition after the first phase optimization: (**a**) distribution of thermal residual stresses within the layers and (**b**) the apparent fracture toughness.

As stated above, the aim of the optimization is to achieve both highest possible values of *K_R,eff_* in the protective layers and at the same time keep the values of *σ_res_* within the layers at a reasonably low level by changing thicknesses and material order of the substrate and protective layers without changing the total thickness of the structure. The optimization process was split into two phases. During the first phase which is schematically shown in Figure 3, the individual materials within outer protective layers and the substrate were changed from ATZ to ZrO_2_. The thickness ratio *h*_1_/*h*_2_ of the outer protective layers was changed in such a manner that either both layers were equally thick or one of them was significantly thicker. The thickness of BaTiO_3_ layers *h_p_* could take values from a discrete set {0.1, 0.15} mm and the thickness of substrate *h_s_* could take values from a discrete set {0.2, 0.3, 0.4} mm. The whole structure was subjected to a temperature change Δ*T* = −1430 °C. This value represents a common temperature difference between the zero-strain temperature and the room temperature [34]. The first phase showed that the best results which are presented in Figure 4, i.e., reasonably low level of thermal residual stresses and sufficiently high apparent fracture toughness in outer protective layers, are achieved when both outer protective layers are equally thick (*h*_1_/*h*_2_ = 1), BaTiO_3_ layers being 0.15 mm thick and ZrO_2_ substrate being 0.4 mm thick. Other combinations lead either to high thermal residual stresses within ATZ and BaTiO_3_ layers or no increase in apparent fracture toughness (*K_R,eff_* was not higher than the intrinsic fracture toughness *K_c,_*_0_ of a particular material) of outer protective layers.

**Figure 5:**
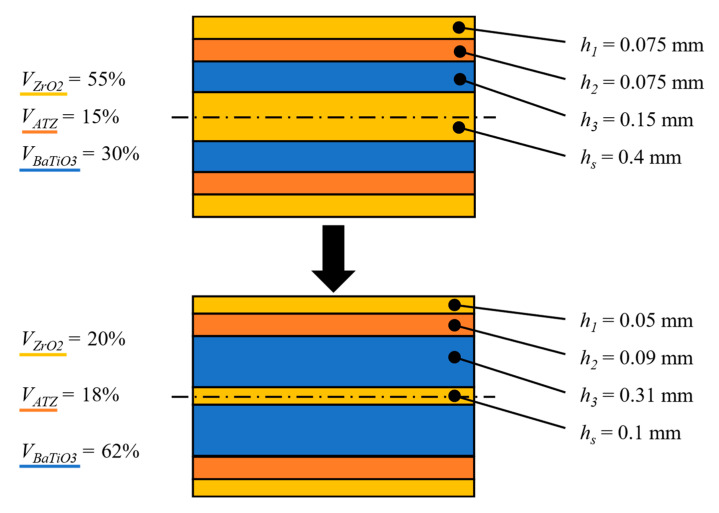
The second phase of optimization showing the input configuration from the first optimization phase (**top**) and the resulting ideal layer composition for the multilayer piezoelectric harvester (**bottom**).

During the second phase of the optimization, the volumetric fractions *V_i_* of used materials were changed so that the BaTiO_3_ layers were as thick as possible in order to reduce their capacitance. The resulting, optimised configuration is shown in Figure 5. ZrO_2_ layers including the substrate were made thinner to allow for thicker BaTiO_3_ layers due to their similar thermal expansion coefficients α.

**Figure 6:**
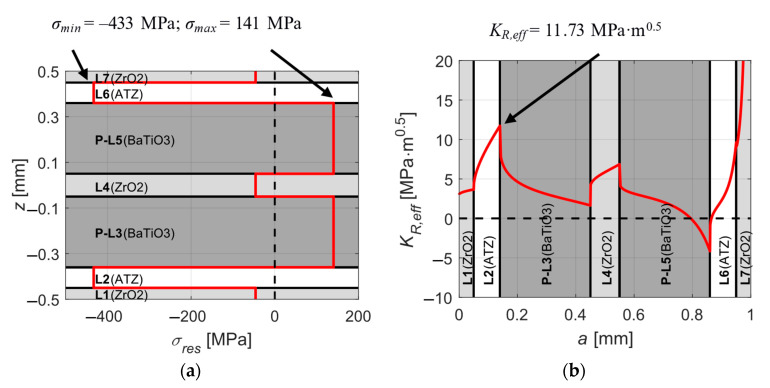
The calculated outcome for the ideal layer configuration loaded with Δ*T* = −1430 °C: (**a**) Distribution of thermal residual stresses within the layers. (**b**) The apparent fracture toughness of the structure.

As depicted in Figure 6a, for a temperature difference of Δ*T* = −1430 °C between zero-strain and room temperature we receive a composition with a relatively low level of tensile residual stresses of 141 MPa within BaTiO_3_ layers and high compressive stresses of −433 MPa in ATZ layers. The Figure 6b shows a significant increase of apparent fracture toughness in ATZ protective layers, compared with the intrinsic value of 3.2, the actual value of 11.73 means almost four-times improved resistance to unstable surface crack propagation. Note that the increase in thickness of BaTiO_3_ layers led to a much higher apparent fracture toughness in ATZ protective layers and substantially lower tensile residual stresses within BaTiO_3_ layers compared with the results from the first optimization phase (Figure 4).

## 3. 1DOF Model of Multilayer Piezoelectric Harvester

To determine the electromechanical response of the multilayer piezoelectric harvester, which is excited by ambient vibration with frequency *ω,* an appropriate computational model must be employed. The chosen dimensions of the considered harvester allow for applying the thin beam (Euler–Bernoulli) theory. Hence, if the beam is forced with a forcing frequency close to its first natural frequency *ω_r_*, its transverse displacement relative to the base *w* can be represented by its first, mass normalised, mode shape ϕ_1_ and a modal coordinate *η* as:

(3) $$w\left(x,t\right)\approx \phi_{1}\left(x\right)\eta \left(t\right).$$

The first natural frequency *ω_r_* can be extracted from the following transcendental equation from [35]:

(4) $$1+\cos\lambda_{1}\cosh\lambda_{1}+\lambda_{1}\frac{M_{t}}{mL}\left(\cos\lambda_{1}\sinh\lambda_{1}-\sin\lambda_{1}\cosh\lambda_{1}\right)=0$$

where *M_t_* is the tip mass and *m* is mass of the composite beam per unit of its length which is simply defined as:

(5) $$m=\sum_{i=1}^{N}\rho_{i}Bh_{i}$$

where *N* is a number of layers and *ρ_i_* is a density of the *i*-th layer. *λ_1_* is then defined as:

(6) $$\lambda_{1}^{2}=\omega_{r}\sqrt{\frac{mL^{4}}{\sum_{i=1}^{N}E_{i}J_{i}}}$$

where $\sum_{i=1}^{N}E_{i}J_{i}$ represents the bending stiffness of the composite beam as a sum of elastic modulus *E_i_* and cross-sectional moment of inertia *J_i_* of *i*-th layer referenced to the geometrical centre of the beam’s cross section. ϕ_1_ can then be extracted from:

(7) $$\phi_{1}\left(x\right)=C_{1}\left[\cos\frac{\lambda_{1}}{L}x-\cosh\frac{\lambda_{1}}{L}x+\varsigma_{1}\left(\sin\frac{\lambda_{1}}{L}x-\sinh\frac{\lambda_{1}}{L}x\right)\right]$$

where *ς*_1_ is defined as:

(8) $$\varsigma_{1}=\frac{\sin\lambda_{1}-\sinh\lambda_{1}+\lambda_{1}\frac{M_{t}}{mL}\left(\cos\lambda_{1}-\cosh\lambda_{1}\right)}{\cos\lambda_{1}+\cosh\lambda_{1}-\lambda_{1}\frac{M_{t}}{mL}\left(\sin\lambda_{1}-\sinh\lambda_{1}\right)}$$

*C*_1_ in (7) represents a modal amplitude constant which should be evaluated from (9) so that the first mode shape is mass normalised:

(9) $$\int_{0}^{L}\phi_{1}\left(x\right)m\phi_{1}\left(x\right)dx+\phi_{1}\left(L\right)M_{t}\phi_{1}\left(L\right)=1.$$

Then, since the considered multilayer harvester can be classified as a bimorph with added protective layers whose piezoelectric layers are connected electrically in parallel, one can utilise the single degree-of-freedom computational model from [35] whose governing equations are:

(10) $$\ddot{\eta }\left(t\right)+2b_{r}\omega_{r}\dot{\eta }\left(t\right)+\omega_{r}^{2}\eta \left(t\right)+\kappa U\left(t\right)=f\left(t\right)$$

(11) $$C_{p}\dot{U}\left(t\right)+\frac{U\left(t\right)}{R_{l}}=\kappa \dot{\eta }\left(t\right)$$

where *b_r_* is a damping ratio, *κ* is the modal electromechanical coupling term, *U* is the voltage drop generated in piezoelectric layers, *f* is the modal forcing function, *C_p_* is the capacitance of piezoelectric layers, and *R_l_* is connected resistive load. *κ* is in [35] defined as:

(12) $$\kappa =-2\cdot e_{31}B\frac{h_{p}+h_{s}}{2}{\frac{d\phi_{1}\left(x\right)}{dx}|}_{x=L}$$

where *e*_31_ is piezoelectric modulus of BaTiO_3_ – $e_{31}=Ed_{31}$, *h_p_* is the thickness of BaTiO_3_ layers, and *h_s_* is the thickness of the substrate. Next, *f* is defined as:

(13) $$f\left(t\right)=A_{0}\left[m\int_{0}^{L}\phi_{1}\left(x\right)dx+M_{t}\phi_{1}\left(L\right)\right]e^{i\omega t}$$

where *A*_0_ is the acceleration amplitude of kinematic excitation. *C_p_* is simply defined as:

(14) $$C_{p}=2\frac{\epsilon_{33}^{S}BL}{h_{p}}$$

where $\epsilon_{33}^{S}$ is a permittivity of the BaTiO_3_ measured at constant mechanical strain and defined as $\epsilon_{33}^{S}=\epsilon_{33}^{T}-d_{31}e_{31}$.

Equations (10) and (11) can be modified for direct calculation of the relative beam’s free end displacement *w*(*L*) by rewriting (3) into:

(15) $$\eta \left(t\right)=\frac{w\left(L,t\right)}{\phi_{1}\left(L\right)}$$

By substituting (15) into (10) and (11) we obtain a following system of ODEs with unknowns being *w*(*L*, *t*) and *U*(*t*)

(16) $$m_{eff}\ddot{w}\left(L,t\right)+b_{eff}\dot{w}\left(L,t\right)+k_{eff}w\left(L,t\right)+\theta U\left(t\right)=-m_{acc}\ddot{z}\left(t\right)$$

(17) $$\dot{U}\left(t\right)=\frac{1}{C_{p}}\left(\theta \dot{w}\left(L,t\right)-\frac{U\left(t\right)}{R_{l}}\right)$$

where $m_{eff}=\frac{1}{\phi_{1}\left(L\right)}$ represents effective mass of the harvester, $b_{eff}=\frac{2b_{r}\omega_{r}}{\phi_{1}\left(L\right)}$ is effective damping, $k_{eff}=\frac{\omega_{r}^{2}}{\phi_{1}\left(L\right)}$ is effective stiffness of the beam, $\theta =\frac{\kappa }{\phi_{1}\left(L\right)}=-\frac{2\cdot e_{31}B\frac{h_{p}+h_{s}}{2}{\frac{d\phi_{1}\left(x\right)}{dx}|}_{x=L}}{\phi_{1}\left(L\right)}$ is effective electromechanical coupling factor,$m_{acc}=m\left(z_{0}\int_{0}^{L}\phi_{1}\left(x\right)dx\right)+M_{t}\phi_{1}\left(L\right)z_{0}$ is acceleration mass coefficient for calculation of effective force from the base acceleration, and $\ddot{z}=A_{0}e^{i\omega t}$ is the base acceleration.

Equations (16) and (17) use relative transverse displacement *w* as a function of time and position on the beam’s centreline to completely describe the centreline’s deformed shape. The 1DOF model uses only the tip displacement, therefore Equations (16) and (17) can be simplified with a substitution *q*(*t*) = *w*(*L*, *t*) where *q*(*t*) is the tip displacement as a function of time. This substitution leads to a common form of (16) and (17) which is used to simulate the electro-mechanical behaviour of a cantilever beam piezoceramic energy harvester:

(18) $$m_{eff}\ddot{q}\left(t\right)+b_{eff}\dot{q}\left(t\right)+k_{eff}q\left(t\right)+\theta U\left(t\right)=-m_{acc}\ddot{z}\left(t\right)$$

(19) $$\dot{U}\left(t\right)=\frac{1}{C_{p}}\left(\theta \dot{q}\left(t\right)-\frac{U\left(t\right)}{R_{l}}\right)$$

Using the ODE system of a coupled electro-mechanical device (18) and (19), a Simulink model was created to simulate the behaviour of a multilayer cantilever beam under base excitation. Calculated coefficients of 1DOF coupled electro-mechanical model used in the simulation are written in Table 2. This model of a novel multilayer structure with crack protective layers can be used to design an extended model of a bistable vibration energy harvester with additional magnets. A similar 1DOF model was explained and experimentally verified in publication [36].

## 4. Design of Auxiliary Magnetic Spring and Model of Bistable Energy Harvester

**Figure 7:**
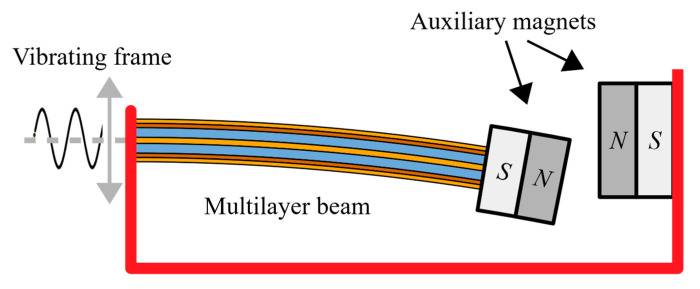
Topology of used auxiliary magnets fixed on the novel multilayer piezoelectric cantilever beam.

The presented well-known system of auxiliary magnets provides a tailor-made design of a kinetic energy harvester based on the novel lead-free cantilever with protective layers. The main aim of this system is to present energy harvesting potential of the novel architecture design with crack protective layers. The used auxiliary magnetic system for a bistable energy harvester consists of an oscillating tip mass permanent magnet and a fixed permanent magnet. The system made use of rare earth magnets FeNB provides a magnetic spring in repulsive direction, which is commonly used to provide nonlinear behaviour to a wider bandwidth of vibration energy harvester. The topology of a bistable piezoceramic energy harvester is depicted in Figure 7. This device consists of the novel BaTiO_3_ piezoceramic layers (Section 2.1) and the auxiliary magnetic system which are analysed and designed for maximal effectivity of harvested power, exactly for the presented BaTiO_3_ architecture.

The presented linear behaviour of ODE coupled electro-mechanical system (18) and (19) is extended by nonlinear magnetic forces of the auxiliary magnetic system in Figure 7. The analysed magnetic force is a function of the tip mass displacement *q*. This magnetic force can be interpreted as a variable stiffness and together with the linear beam’s stiffness *k_eff_*, it is summed with the nonlinear force from magnets, resulting in the total force. This resultant force affects the vibrations of the considered energy harvester and allow for calculation of oscillator’s potential energy via this resultant force.

**Figure 8:**
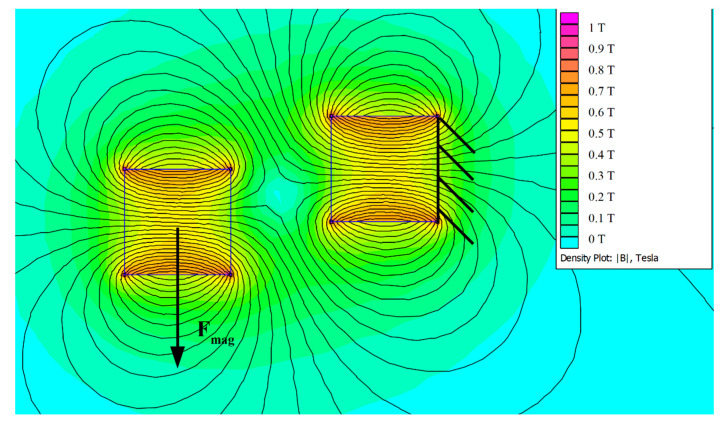
Magnetic Field Simulation of Auxiliary Magnets in Software FEMM 4.2.

Software FEMM 4.2 was used to calculate the magnetic force of the auxiliary magnetic system. This finite element method software can simulate the in-plane magnetic field, and thus calculate the force acting on the magnet. Results of the calculation of the magnetic field are demonstrated in Figure 8. The vertical component of the magnetic force is used in the ODE model (18). The horizontal component of the force is not considered, since it is compensated by the beam’s clamping and it does not affect the oscillations and electro-mechanical conversion.

Dimensions of the permanent magnets and the displacement between them modifies the magnetic force. An important indicator, or one could say a cost function, to design parameters is not this magnetic force, but the total force produced through summation of the magnetic force with the elastic force of the beam (*F_beam_ = k_eff_.q)*. The total force is the main characteristic that might be used to estimate the behaviour of the energy harvester. It could provide stable behaviour with a hardening or softening characteristic, or bistable behaviour.

**Figure 9:**
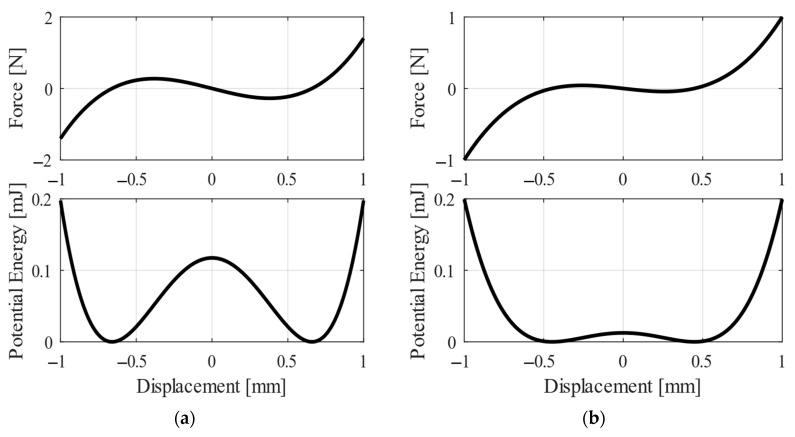
The magnetic force and potential energy in bistable oscillator with (**a**) huge barrier and (**b**) low barrier between two stable positions.

In this research, the bistable behaviour was the goal of auxiliary magnets design. The bistability might provide various operational modes, which are useable in energy harvesting [23]. The barrier between both stable positions is important in bistable design of energy harvester. If the barrier is high, the harvester oscillates around one stable position and does not change to the second one. Lower barrier might often cause repeated swinging between two stable positions. To illustrate the difference, Figure 9 compares a nonlinear oscillator with high and low barrier.

**Figure 10:**
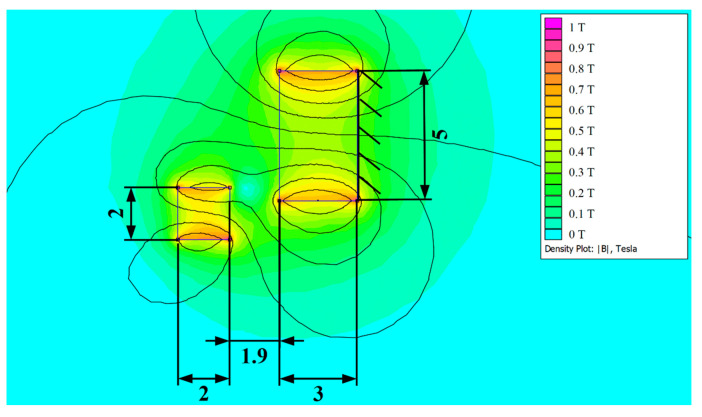
Final design of the Auxiliary Magnetic System; dimensions are in (mm). The depth is 10 mm.

Nonlinear oscillator with low barrier seems to be more sufficient for energy harvesting. Swinging between two stable positions could bring more energy than oscillation around one stable position. Due to this fact, magnets were designed to produce this type of bistability with a low barrier. Design parameters were dimensions of magnets and distance between them. The final design of the magnetic system is demonstrated in Figure 10.

**Figure 11:**
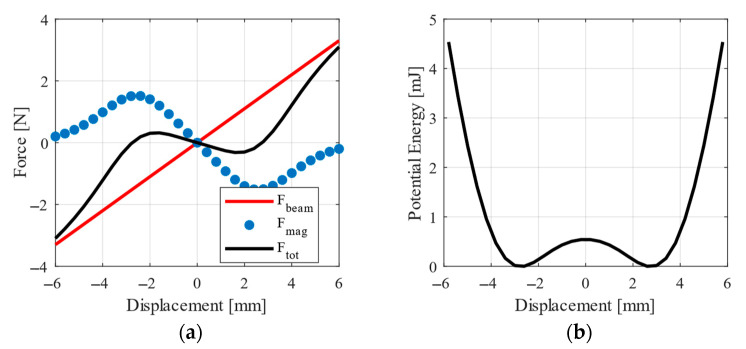
(**a**) Final stiffness force and (**b**) potential energy of the designed oscillator.

These designed auxiliary magnets produce bistable characteristic with low barrier, depicted in Figure 11. Behaviour of the designed system is modelled with Equations (18) and (19) from the previous section extended with the magnetic force represented as a nonlinear function of the tip displacement. The final system is described by these extended equations:

(20) $$m_{eff}\ddot{q}\left(t\right)+b_{eff}\dot{q}\left(t\right)+k_{eff}q\left(t\right)+F_{mag}\left(q\right)+\theta U\left(t\right)=-m_{acc}\ddot{z}\left(t\right)$$

(21) $$\dot{U}\left(t\right)=\frac{1}{C_{p}}\left(\theta \dot{q}\left(t\right)-\frac{U\left(t\right)}{R_{l}}\right)$$

where *F_mag_* is the force from auxiliary magnets, the same as in Figure 11.

## 5. Simulation Results

**Figure 12:**
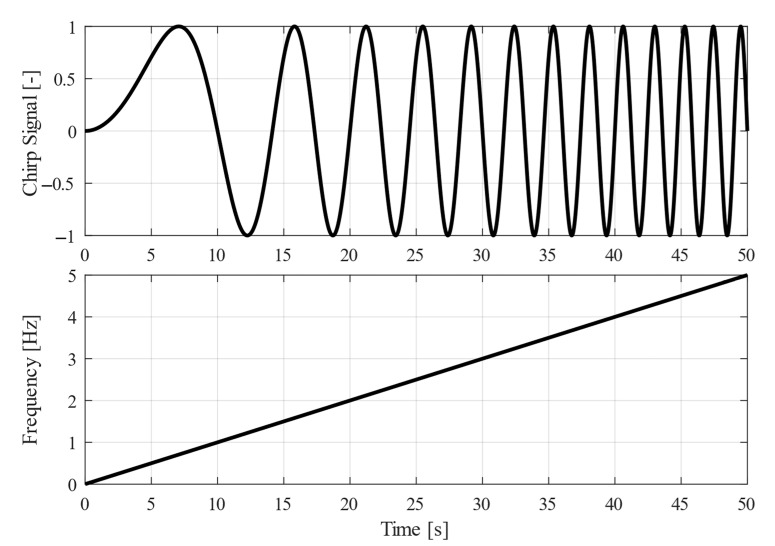
Chirp signal used for simulations.

The designed bistable energy harvesting system of the novel multilayer beam with auxiliary magnets was simulated to estimate its dynamic behaviour and to estimate the crack protection safety. The ambient excitation was modelled in a form of a chirp signal, which is a sine function with a constant amplitude and continuously changing frequency. The chirp signal is demonstrated in Figure 12. Parameters for the simulation are the initial frequency, the final frequency and the speed of change in frequency. The speed of change should be low enough to produce quasi stabilised oscillations. In these simulations, the changing frequency speed was 0.1 Hz/s.

**Figure 13:**
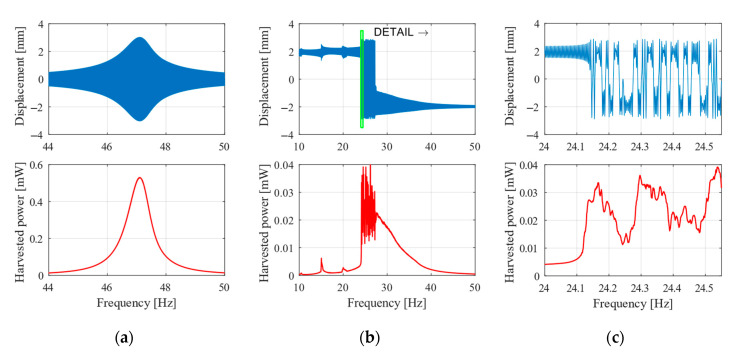
Sweep up with amplitude 0.5 g. (**a**) Linear, (**b**) nonlinear oscillator, and (**c**) detail of nonlinear oscillatations.

The designed vibrational energy harvester (VEH) was tested with varying amplitude of vibrations to estimate the optimal operating range. Figure 13 depicts results from simulation with excitation amplitude 0.5 g, comparing the nonlinear (b) and linear (a) version, and the detail of oscillation between stable positions (c). The displacement plot is a direct tip displacement from the simulations. The harvested power is a moving average power calculated from the actual power on the resistor. The actual power is simply calculated from the voltage on the resistor and the resistance value bas follows:

(22) $$P=\frac{1}{2}\frac{U{\left(t\right)}^{2}}{R_{l}}.$$

The linear energy harvester has a higher power output with a maximum of 0.5 mW for 47 Hz. The nonlinear one has a lower power about 0.03 mW, however, it has a wider frequency range—from 25 to 35 Hz. Depending on the application, the nonlinear energy harvester might be used with an advantage to harvest energy from frequency changes in this range. The linear one can only harvest energy near its resonance of 47 Hz. However, one can say that at this amplitude of vibrations the nonlinear VEH does not bring a significant improvement.

**Figure 14:**
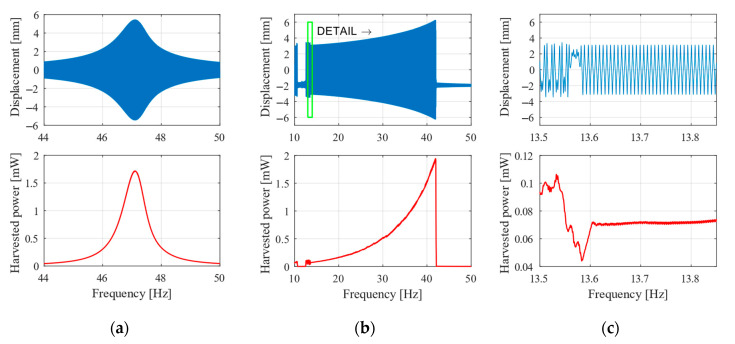
Sweep up with amplitude 0.9 g. (**a**) Linear, (**b**) nonlinear oscillator, and (**c**) detail of nonlinear oscillations.

On the other hand, the situation is completely different with a higher amplitude of excitation acceleration. Figure 14 represents operation with the excitation amplitude 0.9 g. The linear VEH has the same operation frequency 47 Hz and maximal power 1.8 mW. The nonlinear one has a wide operational range from about 20 to 42 Hz, where the power varies from the 0.2 to 2 mW. The maximum power of the nonlinear energy harvester is higher than of the linear one and also the operating range is wider. The nonlinear VEH brings a strong improvement for the amplitude of ambient vibrations: wider operational range and a higher amplitude. However, the important question in this part is the safety of the structure against the propagation of potential surface crack.

## 6. Estimation of Critical Tip Displacement Amplitude

The critical displacement of the beam’s free end is such a displacement amplitude upon which a potential surface crack propagates through the BaTiO_3_ layer, and thus causes a malfunction of the whole multilayer structure. To determine critical displacement amplitude of the harvester’s free end, the stress intensity factor *K_appl_* at the tip of the crack within ATZ protective layers upon vibrations must not be higher than 11.7 MPa·m^0.5^ (see the end of Section 2.1). The stress intensity factor *K_appl_* is defined through the weight function method as:

(23) $$K_{appl}\left(a\right)=\int_{0}^{a}h\left(z,a\right)\sigma_{x0}\left(z\right)dz,$$

where *h*(*z*, *a*) is a weight function defined in [33] and *σ_x_*_0_(*z*) is the amplitude of bending stress distribution within the multilayer structure. The highest values of bending stress upon vibrations consisting solely of the first mode shape are found in the vicinity of the clamping at *x* = 0, which is, therefore, the most suitable location for potential surface cracks. The amplitude of bending stress distribution *σ_x_*_0_(*z*) is, in the case of heterogenous multilayer structure, a layer-wise function and near the clamping at *x* = 0, it can be expressed in individual layers as:

(24) $$\sigma_{x0,i}\left(z\right)=-{E_{i}\frac{d^{2}w\left(x\right)}{dx^{2}}|}_{x=0}\left|z\right|,$$

where subscript *i* denotes the *i*-th layer. Since the bending stress *σ_x_*_0_(*z*) and stress intensity factor *K_appl_* are assumed to vary linearly with the beam tip displacement, one can easily estimate the critical tip displacement amplitude using a safety factor *k_BF_* defined as:

(25) $$k_{BF}=\frac{K_{R,eff}\left(a_{ATZ}\right)}{K_{appl}\left(a_{ATZ}\right)},$$

where *a_ATZ_* is the location of ATZ/BaTiO_3_ interface. If the calculated safety factor *k_BF_* is equal or lower than 1, then a potential surface crack will propagate through both protective layers and cause a brittle fracture of the whole multilayer structure.

**Figure 15:**
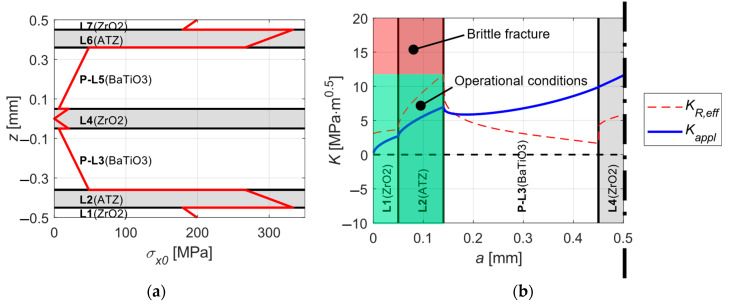
(**a**) Amplitude of the bending stress within the laminate kinematically excited with an amplitude of 0.9 g. (**b**) Stress intensity factor *K_appl_* (blue solid curve) corresponding to excitation amplitude 0.9 g and the apparent fracture toughness *K_R,eff_* (red dashed curve); brittle fracture of the whole structure occurs if *K_appl_* > *K_R,eff_* in ATZ layer, i.e., if the blue solid curve appears in the red-filled area.

For the results given in Section 5. where the harvester is kinematically excited with an acceleration amplitude of 0.9 g and its free end vibrates with a displacement total amplitude of 6 mm the amplitude of the bending stress within individual layers is shown in Figure 15a. The maximal stress values occur in ATZ layers since it is the stiffest material within the laminate. Figure 15b shows the corresponding stress intensity factor *K_appl_* along with the apparent fracture toughness *K_R,eff_* calculated in Section 2.1. The stress intensity factor *K_appl_* reaches the value of 7MPa·m^0.5^ at ATZ/BaTiO_3_ interface which gives a safety factor of 1.67. Therefore, the critical amplitude of the free end’s displacement upon which a brittle fracture occurs is approximately 10 mm. This means a significant increase in resistance against the surface crack propagation compared to a typical harvester from [10] whose critical amplitude of the free end displacement is about 3 mm.

## 7. Discussion

### 7.1. Effect of the Protective Layers

As shown in Section 2, the presented multilayer design of a piezoelectric harvester with high thermal residual stresses significantly increases the resistance to unstable surface crack propagation. In practice, this means that such a multilayer structure can withstand much larger deflections than its traditional counterparts having no protective layers with thermal compressive residual stresses.

On the other hand, as can be seen from Table 1, materials considered for protective layers commonly have higher elastic modulus than piezoelectric materials. If layers with such stiffer materials are too thick it may significantly increase the bending stiffness of the composite beam(which is undesirable for energy harvesting applications, since it lowers the amount of generated electrical power) and contrary when too thin protective layers are considered, the apparent fracture toughness of the structure is increased just slightly [32].

Therefore, the key task is to find a compromise between a very thin beam, which is desirable for energy harvesting applications but very prone to brittle failure, and a beam with thicker protective layers, which increase the beam’s resistance to unstable surface crack propagation but also significantly increase its bending stiffness, and thus lower the amount of generated electrical power.

Design used in this work was optimised to maximise the resistance to unstable surface crack propagation in the presented bistable operational mode. Thanks to this design, the critical tip displacement amplitude was simulated up to 15 mm peak to peak operation without a brittle fracture. Theoretically, the maximal tip displacement amplitude was increased to 10 mm (20 mm peak-to-peak) compared with the tip displacement amplitude of the VEH from [10] without protective layers, for which the cracking occurs at a tip displacement amplitude around 4 mm. The designed BaTiO_3_ beam without the protective layer might have critical tip displacement around 1 mm, which means about 10 times higher crack resistivity with layered architecture than without it.

### 7.2. PZT Solution vs. Proposed Lead-Free Design

In comparison with PZT energy harvesters, the designed lead-free harvester generates lower power due to significantly lower piezoelectric parameters. Commonly used commercial solution Midé V21BL [37] in the resonance operation frequency 40 Hz provides maximal power 0.9 mW upon excitation of 0.5 g. Presented and designed lead-free harvester provides output power around 0.5 mW, operated at 47 Hz for the same excitation of 0.5 g. Dimension of the Midé solution is 64 mm in length with a tip mass 4.8 g. The designed lead-free VEH is 100 mm long with a tip mass 5 g. The resonance frequencies are a slightly different, and this difference could be compensated by longer beam or thinner piezoceramic layers which decrease the natural frequency.

### 7.3. Effect of the Auxiliary Magnets Producing Nonlinear Behaviour

The nonlinear behaviour produced by the auxiliary magnets allows to bring a wider operating bandwidth and a higher maximal power. It is obvious that the linear energy harvester operates in one frequency with various amplitudes of excitation vibrations, whereas the nonlinear one operates in a threshold amplitude of excitation vibrations for a wide bandwidth, but for a lower amplitude, so its operation is unsuitable for the energy harvesting, since the generated power is very low.

## 8. Conclusions

In this article, the idea of crack protective layers was used to design the lead-free cantilever beam, which allows for higher amplitudes of oscillations, and thus to increase also the harvested power. The common lead-free BaTiO_3_ bimorph cantilever can only operate in low amplitudes of the excited oscillations due to a very brittle behaviour. The proposed design of a lead-free cantilever with protective layers allows to use this design for operation in an extended bistable system with significantly higher amplitudes. Due to the implementation of the proposed cantilever design, the auxiliary magnets were used to produce the energy harvesting system with two extended equilibrium distance of bistable system. Furthermore, the stress intensity factor at the tip of potential surface crack in the system, calculated for the maximal displacement demonstrates that the designed architecture is more resistant to surface crack propagation in the simulated operational range.

Designed and presented bistable kinetic energy harvester has lower electric outputs than commercial PZT energy harvesters. However, used BaTiO_3_ material is lead-free, which is very important recently. Moreover, the proposed design is improved by the protective layers allowing higher excitation amplitudes for which the power is just 50% lower than at commercial PZT solutions.

Manufacturing of the proposed composite structure is now under development and the manufacturing process and characterization of materials are published in papers [4,38,39]. The main aim of our future development is the realization of the experiment and to comspare obtained experimental results with other bistable PZT energy harvesting system.

The presented design of a bistable nonlinear system provides operation in a wide frequency bandwidth for an excitation amplitude close to 1 g. The effect of the nonlinearity is disputable for the low amplitude of vibrations. However, for the excitation amplitude around 1 g, the operational frequency bandwidth is in range from 20 to 40 Hz, what can be useful in some engineering application. The harvested power lies in the range between 0.2 and 2 mW, depending on the excitation frequency. The highest power of 2 mW could be used e.g. to power up some remote sensors with wireless data transfer. The presented approach brings this bistable energy harvesting technology near to its application in a data acquisition.

## Figures and Tables

**Table 1:** Mechanical and piezoelectric properties of used materials [24,28,29].

Material		*ρ* (kg/m^3^)	*E* (GPa)	*ν* (–)	*α* (ppm/K)	*K_c,0_* (MPa·m^0.5^)	*d*_31_ (C/N)	$\epsilon_{33}^{T}/\epsilon_{0}$ (–)
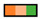	ATZ	4050	390	0.22	9.8	3.2	–	–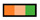	ZrO_2_	5680	210	0.31	10.3	3	–	–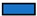	BaTiO_3_	6020	70	0.22	11.5	0.7	–58·10^-12^	1250

**Table 2:** 1DOF model parameters of the considered multilayer beam.

*m_eff_* (g)	*b_eff_* (Ns/m)	*k_eff_* (N/m)	*m_acc_* (g)	*C_p_* (nF)	*θ* (μN/V)	*R_l_* (kΩ)
6.3	3.75·10^-2^	555.8	7.1	75.6	245	45
